# Electromagnetic Fields, Electrical Stimulation, and Vacuum Simultaneously Applied for Major Burn Scars

**DOI:** 10.3390/bioengineering12020179

**Published:** 2025-02-13

**Authors:** Salvatore Marafioti, Sheila Veronese, Claudio Pecorella, Carlo Felice Tavernese, Sara Costantino, Maurizio Busoni, Andrea Sbarbati

**Affiliations:** 1 Marafioti Medical Clinic, Taurianuova, 89029 Reggio Calabria, Italy; salvatore.marafioti@gmail.com; 2 Department of Neuroscience, Biomedicine, and Movement Sciences, University of Verona, 37134 Verona, Italy; andrea.sbarbati@univr.it; 3 Italian Football Federation (FIGC), 00198 Roma, Italy; cpecorella@yahoo.it; 4 Tavernese Dentistry & Medicine, 89042 Reggio Calabria, Italy; cftavernese@gmail.com; 5 Costantino Center, 89125 Reggio Calabria, Italy; saracostantino89@hotmail.it; 6 School of Pharmaceutical and Health Product Sciences, Camerino University, 62032 Macerata, Italy

**Keywords:** electromagnetic field, electrical stimulation, V-EMF therapy, major burn, scar, fibrosis, regeneration, motor function

## Abstract

Background: Regeneration in the case of major burn subjects must involve tissue and structural regeneration, but also functional regeneration, as scars derived from burns often compromise motility. Electromagnetic fields and electrical stimulation may be a possible treatment for these cases, considering they cause a thermal effect and magneto-mechanical transduction first and selective tissue stimulation second. Methods: A case of a majorly burned woman with severe motor deficits, treated with electromagnetic fields and electrical stimulation in vacuum, associated with a personalized nutritional program, was described. The latter was necessary to favor weight loss with the preservation of the weakened structure. Ultrasonography, Doppler ultrasound, and body composition were measured. Moreover, postural evaluation was performed. Results: Immediately after the treatment, a restructuring of all tissue was seen. After 6 months, the tissue regeneration was evident, with neo-angiogenesis. From the functional point of view, her motility improved, and she stopped using a walker. Conclusions: The combined therapy allows her to obtain unthinkable results in a short time. For this reason, it could become the elective treatment for major burn scars.

## 1. Introduction

Electromagnetic fields have been widely used in all medical fields, particularly physiatric treatments. Radiowaves, from very low (<1 Hz) to extra high (300 GHz) frequencies, are intensely applied because of their double effect: the diathermic effect [1,2,3] and the magneto-mechanic effect [4].

The first effect consists of the transformation of the kinetic energy of the electromagnetic wave ions into heat, according to the Joule effect [5,6,7]. It is essential to underline that heat production is endogenous; that is, it is generated inside the tissues [8]. The benefits correlated to thermotherapies have been widely described in the literature [9,10,11].

The second effect causes a realignment of the fibers/cells, according to their iron content, and a behavioral response in other para-magnetic substances [4]. Moreover, this effect is strongly correlated to the piezoelectric activation of tissues, that is, to their ability to mechanically alter their structure following magnetic stress [12,13]. Collagen is one of the body’s tissues with higher piezoelectric properties [14], and its response to the magneto-mechanic effect is its lengthening and expansion [15] and a consequent extracellular matrix (ECM) restructuring [14].

The tissues’ response is correlated with the specific frequency range of the electromagnetic field applied. The resistive capacitive radiofrequency has a range of 0.3–3 MHz. Its generators may work in a capacitive or resistive way. The capacitive system is highly effective on muscles and soft tissues, while the resistive system is suitable for tendons and bones. Often, these two systems are used in a combined protocol to reduce chronic pain conditions [15,16,17,18,19,20,21] and improve the functional deficits associated with the pain [18,19,20,21,22], leading to an improvement in the quality of life (QoL) of the treated subjects [18,19,21].

The capacitive radiofrequency alone has been applied in the aesthetic field to induce a morphological change at the dermis and hypodermis levels in the orientation of collagen and elastic fibers. At 0.55 and 1 MHz, it was successfully applied to cellulite to reduce its typical dimpled appearance [23,24]. Their effects on collagen and elastic fibers have been tested at 0.52 MHz in the abdominal region [25] and 0.3–0.5 MHz in the back [26]. In this last study, neo-collagenesis and neo-angiogenesis, associated with an increase in the thickness rate of the dermis, were reported.

The combination of a capacitive radiofrequency with a range of 0.5–2 MHz with vacuum and electrostimulation, a combination known as V-EMF therapy, is fascinating. This therapy has been successfully applied to different aesthetic problems, but mainly for reducing the appearance of scars [27,28,29] and stretch marks [30,31] and as an anti-aging treatment, considering that it improves skin laxity [32,33].

This therapy has been particularly effective in all the conditions in which an alteration of the collagen and elastic fibers was present. In addition to the effects of capacitive radiotherapy, it exploits the vacuum and electrostimulation effects.

The literature analysis revealed that vacuum is utilized alone as a massage or associated with other treatments to enhance clinical outcomes [34]. It seems to promote ECM production [35], improving skin distensibility and viscoelasticity [36]. Combined with other treatments, it has been applied for contouring therapies to reduce adipose tissue depots and cellulite [37,38,39,40]. In a very interesting study, Moortgat et al. [41] highlighted how the results of this treatment are related to its parameters (i.e., duration of application, amplitude, and frequency of stimuli). This lets us understand that an optimization correlated to all the specific problems treated has yet to be achieved.

Electrostimulation has been widely used since the 1700s. Nowadays, it is employed mainly to vehiculate ionized drugs through the skin, to reduce pain, and to stimulate particular tissues. The first use is also known as iontophoresis or electrophoresis [42,43]. The second one exploits the membrane hyperpolarization to increase the excitability threshold of nerves and nociceptors and the gate control analgesic mechanism [44,45]. The last one induces the selective stimulation of nervous fibers, the muscular tissue innervated by these fibers, and the denerved muscular tissue [46,47,48,49].

The whole effect of V-EMF therapy has resulted in a restructuring/regeneration of the tissues treated and pain reduction, always without discomfort during treatment application and without subsequent side effects.

In Veronese et al. [29], the case of a woman with major burns was presented, and the improvement of some functional aspects after V-EMF therapy was highlighted (skin sensitivity, face and neck movement, respiratory and masticatory function). This was the first case in which this therapy was applied to a so widely diffused scar, and it is the only one present in the literature. In this new study, the case of another woman with major burns and severe functional impairment was presented. V-EMF therapy was associated with a personalized nutritional program to favor functional recovery. The aims of this study are to verify the functional improvement of the woman with major burns correlated to the effect of the V-EMF therapy, to discuss the regenerative mechanisms of the V-EMF therapy combined with the nutritional program, and to evaluate the QoL improvement of this woman, secondary to the functional benefits.

## 2. Materials and Methods

### 2.1. Case

The woman described in this study was 45 years old at the time of the treatments. Four years previously, she was the victim of a domestic assault with petrol, resulting in severe burns over 80% of her body. From the assault to the V-EMF therapy application (April 2023), she had undergone more than 200 surgeries and long-term hospitalizations.

At the beginning of 2023, she presented a diffuse compromise, both from the aesthetic and functional points of view.

**Figure 1:**
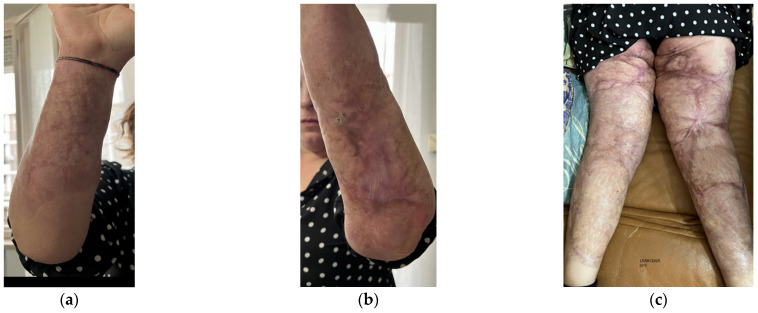
Details of the burns results. (**a**) Diffuse fibrotic scar on the right forearm; (**b**) Diffuse fibrotic scar on the left forearm with skin retraction and tissue depression; (**c**) Both the legs presented severe wide scars, fibrotic areas, skin retraction, areas of tissue depression, keloids, hypertrophic scars, and pigmentation disorders. The aesthetic problems were strictly correlated to functional motor problems.

Aesthetically, she had severe wide scars, fibrotic areas, skin retraction, areas of tissue depression, keloids, hypertrophic scars, and pigmentation disorders (Figure 1). She reported spontaneous pain, managed by drugs.

Functionally, she presented severe motor deficit with incomplete knee flexion, tissue stiffness of both upper and lower limbs, sensitivity absence, hair absence, sweating absence, superficial vascularity absence, and strongly compromised skin elasticity. She used a walker to walk.

### 2.2. V-EMF Therapy

Twelve sessions of the V-EMF therapy were administered from April to July 2023, according to the Biodermogenesi^®^ protocol. The treatment was applied by the Bi-one^®^ LifeTouchTherapy device (Expo Italia Srl, Florence, Italy). This device has two microprocessors. The first one activates, triggers, and stops the stimulations, that is, the patient’s treatment. It controls a radiofrequency generator, an electric pulse generator, and a vacuum pump and includes a biofeedback control system. The second one continuously monitors the first microprocessor’s functionalities. The treatment is delivered through a specific handpiece directly connected to the first microprocessor.

The radiofrequency generator emits an EMF. The generator is a capacitive system with a capacitor composed of a 1st type of armature covered with electrically insulating material and a 2nd type of armature consisting of a return electrode and the body’s tissues. As the body is not a conductor nor an insulator, and the skin is composed of different non-homogeneous layers, a water-based cosmetic is used to favor the waves’ diffusion and a disposable non-cytotoxic PVC cover (ISO 10993) [50] was applied to insulate the electrode, creating a perfect capacitive system. The frequency ranged from 0.5 to 2 MHz, and the average power was 4–6 MHz. The temperature output ranged from 39 °C to 40 °C. The frequency range is different with respect to those used in resistive–capacitive diathermic treatments, which is 0.4–1.2 MHz. The use of a higher frequency permits the concentration of the EMF on the surface tissues, avoiding a too-deep diffusion, according to the fact that the penetration is inversely correlated to the frequency.

The biofeedback control system permits the monitoring of the EMF penetration. Artificial intelligence (AI) software defines the depth of the lesion and the electromagnetic and electric conductibility of the underlying tissues on the basis of the patient’s telemetry. Thus, it establishes the stimulation intensity and frequency. The aim of this stimulation is to increase the temperature, avoiding dangerous tissue overheating.

According to the AI control, the electric pulse generator emits a 5 Hz square wave with an output of up to 0.36 mA at 500 Ohm. The use of this stimulation permits both vehiculating nutritional elements into the tissue, which are contained in cosmetics applied to facilitate the handpiece scrolling, and producing an analgesic effect, which explains the pleasant sensation reported by the patients.

The radiofrequency and electric pulse generators are mechanically, galvanically, and optically isolated.

The vacuum pump provides a negative pressure ranging from 100 to 130 mbar. The negative pressure amplifies the effects of the applied EMF and electric current without a temperature alteration.

The handpiece used for the treatment is externally covered by a high-level fireproof certified resin (Class 0). The base presents an external ring containing a group of electrodes for electrostimulation with rotation of the polarity (ISO 5832) [51]. Internally, it presents an EMF generator shielded disk. Between the ring and the disk, it presents a chamber of air for vacuum production (ISO 10993).

If the return electrode detaches from the skin, the device automatically locks, preventing any unwanted current leakage and, consequently, operating in total security. Moreover, the intensity of the EMF and the applied currents are in a range that permits the delivery of the treatment in a common environment (not shielded) and without particular personal protective equipment for both the operator and the patient. Finally, for the same intensity limits, the patient can return to his/her everyday life habits after the treatment.

### 2.3. Nutritional Program

During the same period, the patient received the V-EMF therapy and she followed a personalized nutritional program as her initial BMI was 31.8 kg/m^2^. The obesity state correlated with the structural deformity contributed to worsening the functional impairment. The weight loss was necessary to ameliorate the body’s functionality, but the structure of a burned subject is generally weak [52]. For this reason, a personalized program was created and applied to help the patient lose weight without further weakening the structure.

The nutritional intervention was structured around a six-month cyclical approach, utilizing ketogenic meals provided by Food Italia Group (Manoppello, PE, Italy). Specifically, the protocol alternated between a 21-day ketogenic phase, referred to as the “Very Low-Calorie Ketogenic Diet (VLCKD) Protocol”, and a 20-day phase of a subject-specific low-carbohydrate diet. Both phases incorporated carefully designed ketogenic meals to ensure compliance and optimize metabolic and therapeutic outcomes.

### 2.4. Imaging Analysis

Before the first treatment (T0), 1 week after the end of the whole treatment (T1), and 6 months after the end of the treatment (T2), ultrasonography and Doppler ultrasound were performed to evaluate structural variations correlated to the therapy. Four areas were tested as reference areas: forearm, popliteal fossa, calf, and posterior thigh, both right and left.

A Samsung HS70 ultrasound device with the LA3-16A linear probe (Samsung Healthcare Italia, Milan, Italy) was used for these evaluations.

### 2.5. Body Composition and Functionality Analysis

Whole body and segmental measurements were assessed using a segmental bioelectrical impedance analysis (BIA) device at 50 KHz (Quantum V Segmental BIA-RJL Systems, Milan, Italy), and results were processed by the manufacturer’s software.

Representative avatars for 3-dimensional (3D) postural evaluation were obtained through the Fit3D ProScanner system (Fit3D, San Mateo, CA, USA), 5.0.6 hardware version and 5.5.0 software version.

The assessments were performed at the exact times as imaging evaluations before (T0) and after (T1) the treatment and at the 6-month follow-up.

## 3. Results

### 3.1. Basal Imaging Evaluations

**Figure 2:**
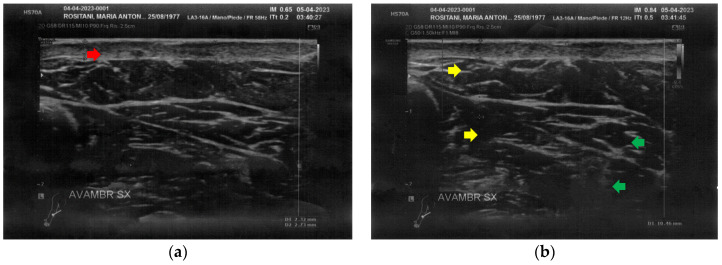

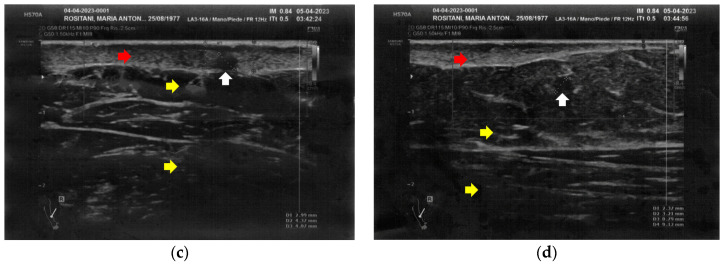
Ultrasonography of the forearms at T0. (**a**) Cicatricial fibrosis (red arrow) of the left forearm involves the whole dermal layer; (**b**) The inflammation (yellow arrows) is extended down to the deeper layer and, in particular, it involves the surgical grafts (green arrows); (**c**) Cicatricial fibrosis (red arrow) is also evident on the right side, where the adipose tissue appears inhomogeneous (yellow arrows) and where a cicatricial granuloma is present (white arrow); (**d**) The aspect described in Figure 2c is similar in other areas of the right forearm, where other cicatricial granulomas are present (white arrow).

At T0, both forearms showed diffuse cicatricial fibrosis involving the entire thickness of the dermis and the upper hypodermis layer, with a diffuse inflammatory state down to the deeper layers, where tissue heterogeneity was evident. The severity of fibrosis was more significant on the right side, where two cicatricial granulomas were also found (Figure 2).

**Figure 3:**
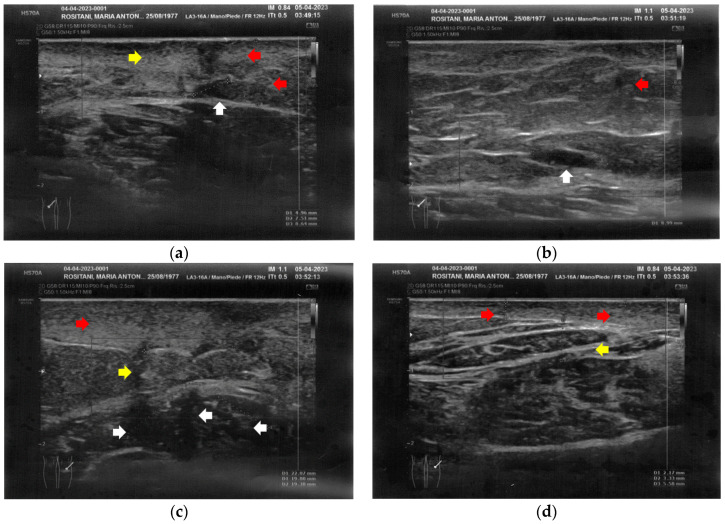
Ultrasonography of the popliteal fossae at T0. (**a**) Wide section of cicatricial fibrosis (red arrow) of the left popliteal fossa with absence of clear anatomic layers (yellow arrow) and presence of a cicatricial granuloma (white arrow); (**b**) In another area of the left popliteal fossa the inhomogeneity of the tissues is evident (yellow arrow), and another cicatricial granuloma (white arrow) is present; (**c**) Cicatricial fibrosis (red arrow) is also evident on the right side, where the adipose tissue appears inhomogeneous (yellow arrows) and where multiple cicatricial granulomas (white arrow) are present in the deeper layer; (**d**) In another area of the right popliteal fossa, the superficial fibrosis (red arrows) appear less thick, and a partial restructuring of the deeper anatomical layers is noted (yellow arrow).

Similarly to the forearms, the popliteal fossae presented diffuse cicatricial fibrosis involving all the tissues down to the upper hypodermis layer. Even in these areas, cicatricial granulomas were present bilaterally, in correspondence to areas of previous abscesses, due to post-operative infections with rejection of the sutures and diastasis of the wounds. On the right side, some areas of minimal extension presented a partial inhomogeneous structural reorganization of the layers (Figure 3).

**Figure 4:**
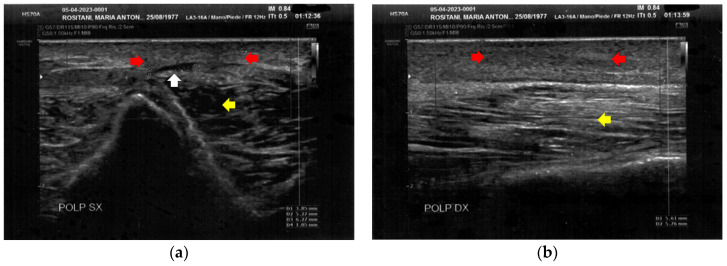
Ultrasonography of the calves at T0. (**a**) Cicatricial fibrosis (red arrows) is evident on the left calf on the superficial layers. Phlogosis extends to the deeper layers (yellow arrow). A small fluid element (white arrow) is observed at the muscular level; (**b**) Cicatricial fibrosis (red arrow) is also evident on the right side, where the muscular structure (yellow arrow) appears preserved.

The left calf presented more severe alterations compared to the right calf. Both areas presented superficial cicatricial fibrosis. However, on the left side, a small fluid element of a probably phlogistic nature was observed at the muscular level, while on the right side, the muscular structure appeared intact (Figure 4).

**Figure 5:**
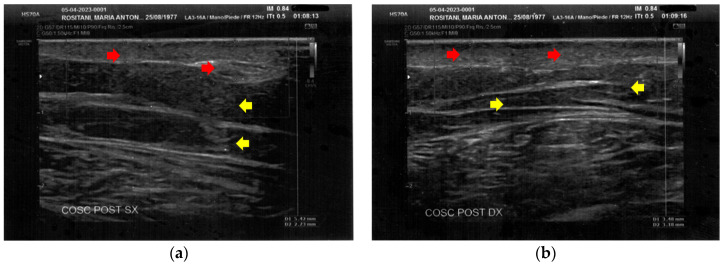
Ultrasonography of the posterior thighs at T0. (**a**) On the left thigh, cicatricial fibrosis (red arrows) is evident on the superficial layers, and phlogosis extends to the deeper layers (yellow arrows); (**b**) On the right thigh, cicatricial fibrosis (red arrows) is present as on the left side, and the deeper layers present an inhomogeneous restructuring (yellow arrows).

The posterior thighs presented diffuse cicatricial fibrosis and a destructuration of deeper layers (Figure 5).

In all the areas tested, superficial vascular elements were absent.

### 3.2. Imaging Evaluations After the Treatment

**Figure 6:**
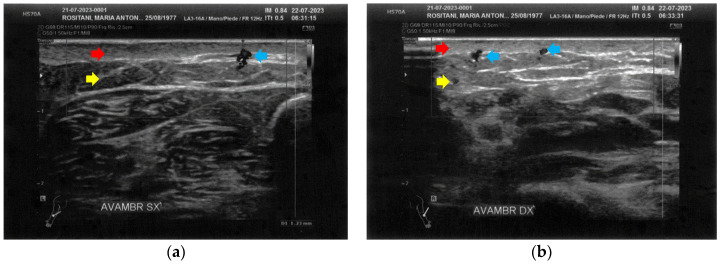
Ultrasonography of the forearms at T1. (**a**) A significant reduction of the cicatricial fibrosis (red arrow) is evident on the left forearm. A restructuring of all layers is also appreciable (yellow arrow). Moreover, some superficial vascular elements (blue arrow) are present; (**b**) The right forearm presents the same ameliorations described for the left forearm.

At T1, both forearms showed a clear improvement of all the damaged tissues, with a reduction of fibrosis and disappearance of cicatricial granulomas. A restructuring of the layers was also evident. Of paramount relevance was the appearance of superficial vessels (Figure 6).

**Figure 7:**
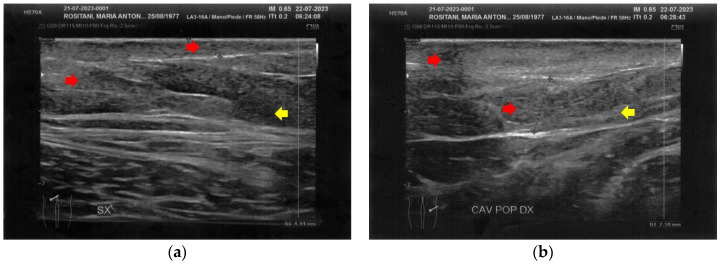
Ultrasonography of the popliteal fossae at T1. (**a**) The left side presents a clear improvement in both the cicatricial fibrosis (red arrows) and the reorganization of the different structural layers (yellow arrow); (**b**) The right side presents the same effects of the treatment noted for the left side.

**Figure 8:**
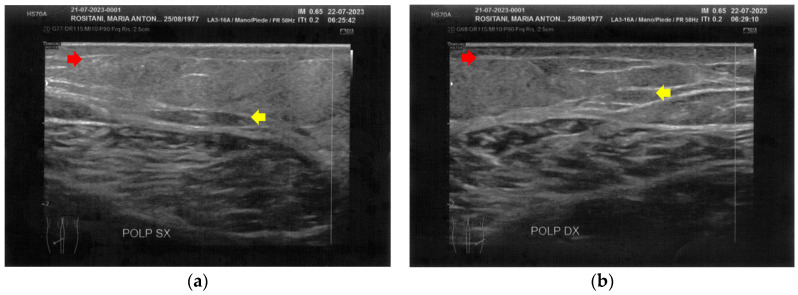
Ultrasonography of the calves at T1. (**a**) The left side presents a clear improvement in both the cicatricial fibrosis (red arrow) and the reorganization of the different structural layers (yellow arrow). The muscular structure appears clearly defined, with a net improvement concerning the initial condition; (**b**) The right side presents the same treatment effects noted for the left side. On this side, the muscular structure was less compromised than that of the left side. The treatment acts on the connective tissue, softening the structure.

**Figure 9:**
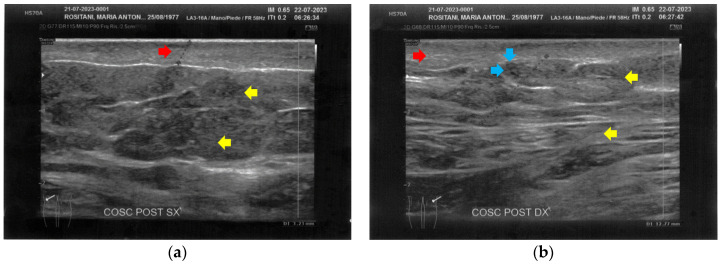
Ultrasonography of the posterior thighs at T1. (**a**) On the left side, an improvement of the cicatricial fibrosis (red arrow) is clear. A restructuring of all the layers (yellow arrows) is also present; (**b**) The right side presents the same improvements as the left. The appearance of superficial vascular elements (blue arrows) is evident.

Like the forearms, the popliteal fossae, the calves, and the posterior thighs presented a clear improvement of the damaged tissues and a restructuring of the layers (Figure 7, Figure 8 and Figure 9). On the right posterior thigh, the appearance of superficial vessels was detected (Figure 9).

**Figure 10:**
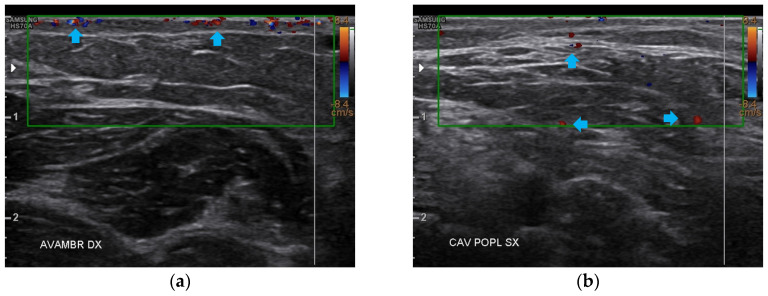

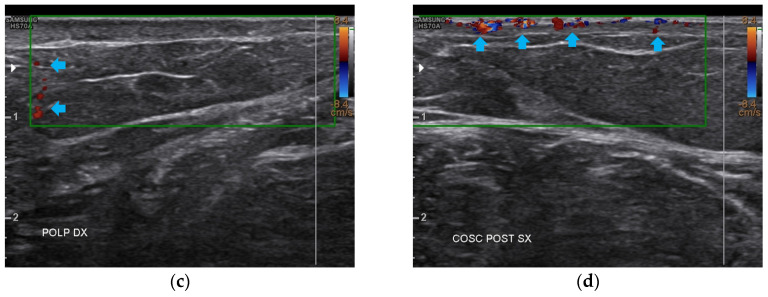
Doppler ultrasound of the four areas analyzed. (**a**) Right forearm; (**b**) Left popliteal fossa; (**c**) Right calf; (**d**) Left posterior thigh. All the areas present a clear revascularization (blue arrows) extending to the deeper layers, particularly on the popliteal fossae and calves.

At T2, all four anatomical areas bilaterally presented an evident reduction of fibrosis and the restructuring of the typical layers. The appearance of both a well-diffused superficial and deep vascularization was also noted (Figure 10).

### 3.3. Nutritional Program Effects, Body Composition, and Functionality Analysis Pre- and Post-Treatment

The parameters related to the body composition are reported in Table 1. Since the first follow-up, weight loss and a corresponding body mass index (BMI) reduction were evident. The body was dehydrated at T0, and at T2, the total body water had increased and resulted in a normal range, with a change in the water distribution across the body (reduction of the extra-cellular water and increase in the intra-cellular water). The fat-free mass (FFM) and the mineral mass (MM) increased, while the fat mass (FM) reduced. The basal metabolic rate (BMR) and the total daily energy expenditure (TDEE) increased.

These parameters were associated with a generalized reduction in body circumference (Table 2).

**Figure 11:**
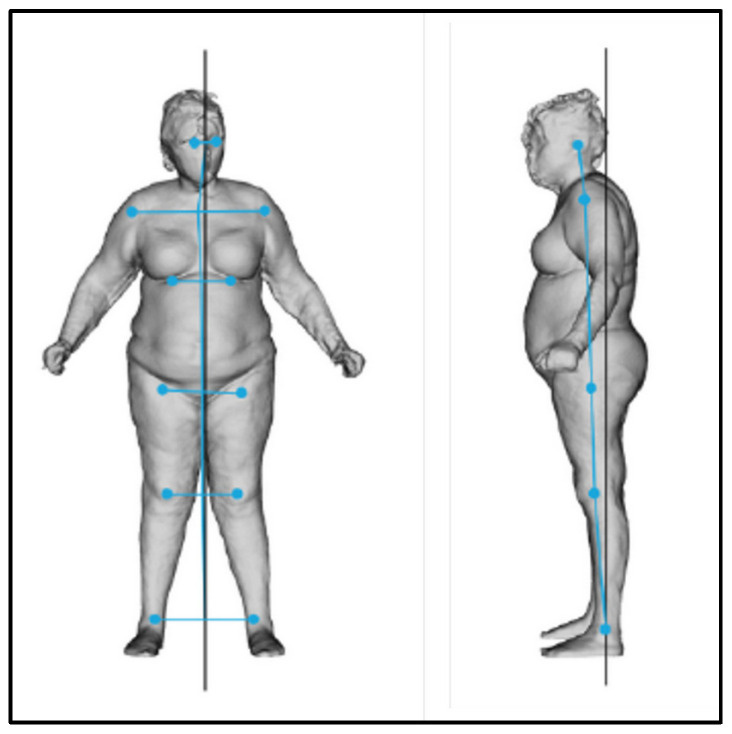
Frontal and lateral postural evaluation.

At T0, the postural evaluation revealed an overall imbalance of the body to the right and forward (Figure 11, Table 3 and Table 4).

**Figure 12:**
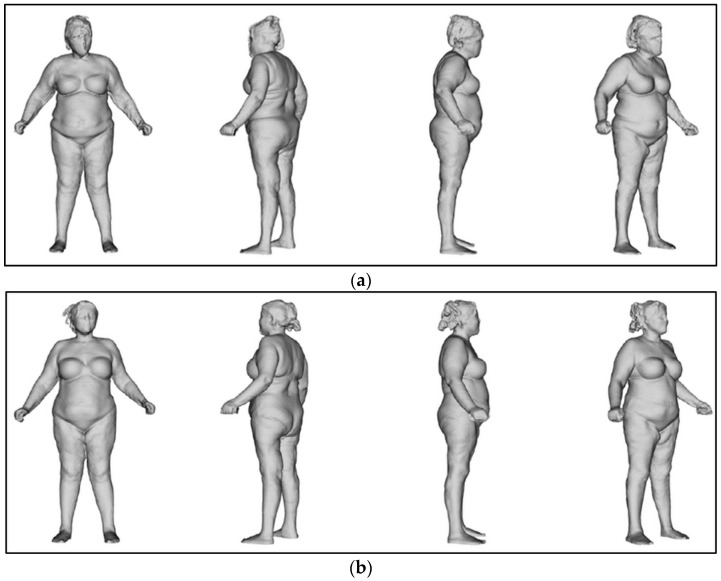
Postural evaluation. (**a**) At T0, the body shifted to the right and leaned forward. (**b**) At T2, the body was perfectly in line.

The body was perfectly aligned after the treatments (Figure 12).

### 3.4. Overall Results

**Figure 13:**
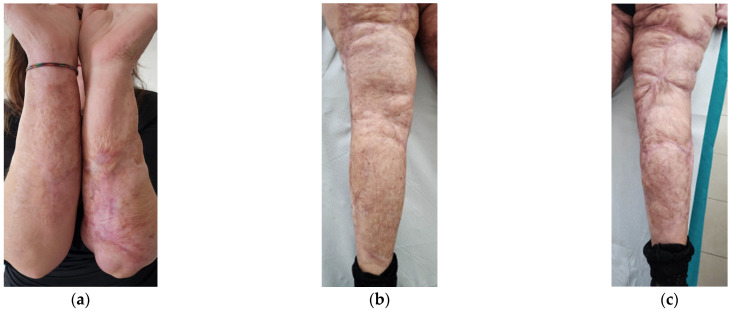
Details of the scars at T2. (**a**) Forearms; (**b**) Left leg; (**c**) Right leg. In all the areas, fibrosis is reduced. Skin retraction and tissue depression are still present only in limited regions. The aesthetic improvement is correlated to functional motor improvement.

After the treatments, a well-defined reduction of scar severity was noted, with resolution of fibrotic and inflammatory conditions. Skin retraction and tissue depression almost completely disappeared (Figure 13). The woman reported that she had stopped using painkillers. Moreover, she felt aesthetically better and had an increase in her self-esteem.

**Figure 14:**
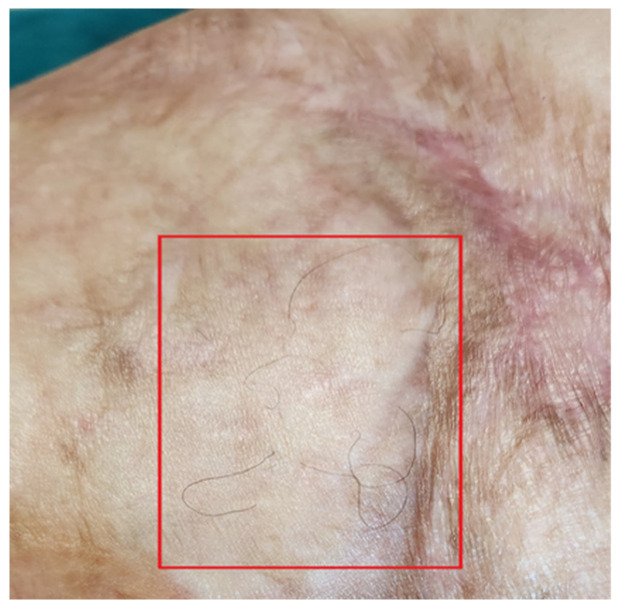
Detail of hair regrowth on the leg.

Functionally, her severe motor deficit reduced. Limbs’ motility improved, and she started to walk without using the walker and could run a few meters at a light pace. The reappearance of skin sensitivity and sweating was reported. The complete restructuring of some areas was noted. The appearance of hair on the legs was documented (Figure 14).

## 4. Discussion

The capability of V-EMF therapy to restore functional aspects has already been documented [27,28,29,32]. In particular, the normalization of different skin parameters, denoting skin functionality recovery, has been observed in all these studies. Hydration levels normalized [27,28,29,32], as well as sebum quantity, pH [29,32], and elasticity [27,29]. In all these studies, the treatment was principally related to aesthetic problems, such as scars and stretch marks. Although it has been proven that these skin alterations compromise not only the skin surface but also the deeper anatomical layers, hypodermis in particular [53,54,55], the studies of Nicoletti et al. [27], Veronese et al. [28], and Laura et al. [32] refer to localized alterations. Instead, the study of Veronese et al. [29] was related to a diffuse face scar that induced a severe functional alteration in the subject treated. The resulting increased motility of the face and neck had improved masticatory and respiratory functions, with a consequent improvement in the QoL of the woman, the subject of the study. In this study, the functional outcomes were reported but not documented with measurements and imaging evaluations.

The question that arose was how the treatment had contributed to improved functionality. The restructuring of elastic and collagen fibers, well documented by Scarano et al. [31], could not alone demonstrate these results. The ultrasonography evaluations performed in the present study highlight the progressive restructuration of all the anatomical layers of the limbs.

Fibrosis progressively resolves. But the interesting aspect is that, at T0, fibrosis involved both the dermis and hypodermis, forming an entire plane of deep thickness, in which the two different structures were indistinguishable. Consequently, the resolution of fibrosis implied, first of all, an increase in cellular metabolism and catabolism and, secondly, an increase in cells’ proliferation with complete regeneration of the ECM.

The first effects correlate to the thermal effect (Joule effect). The increase in metabolic reactions [56,57] determines an increase in microcirculation [5,6,7] and the number of gaseous exchanges between blood and tissues. The catabolic products are drained more quickly, and anti-inflammatory and reparative processes increase [58,59]. Moreover, the thermal effect increases the “cell-killing” effect of senescent and damaged cells [60].

The second effect is strictly correlated to the magneto-mechanical effect (MME). Both cellular proliferation and clearance are promoted, thanks to the transformation of the electromagnetic force in mechanical stimulation of the tissues [4,61,62,63]. Moreover, the MME strongly correlates to the piezoelectric activation of tissues, particularly connective tissue, in the ECM [12,64,65,66]. This activation corresponds to the repair of ECM alterations [63,64] and regeneration of the structure [14,67].

It is impossible to talk of “tissue regeneration” if all the tissue’s aspects are not entirely regenerated. The regeneration must include a vascular repair if vessels are damaged or neo-angiogenesis if absent. The Doppler analysis performed in the present study revealed the formation of a complete vascular net. Electromagnetic fields can promote and modulate neo-angiogenesis [68,69], favoring tissue regeneration. Additionally, the nervous components must also be regenerated. This is crucial in maintaining the effects of the treatments over time, even through retractive feedback to promote regeneration, as the new nerves can send messages to the brain to enhance the healing phase. Nerve repair and regeneration are promoted/mediated by electromagnetic fields [70,71,72] and electrical stimulation [73,74].

In addition to electromagnetic fields and electrical stimulation, V-EMF therapy applies a continuous vacuum massage. It has already been stated that vacuum enhances the effects of other treatments [34] and promotes ECM formation [35]. For this particular case, even its action on fat is paramount. Vacuums associated with different technologies result in highly efficient fat reduction [38,39,40,75].

In this case, the profound alteration of all the tissues induced a severe compromise of all the motor functional aspects. Consequently, the regeneration of the structure, complete with vascular and nervous regeneration, had to be associated with a restoration of the motor function. The presence of an obesity state made functional recovery more complex and risked compromising tissue regeneration since it is known that a state of immobility weakens the structure, in particular the muscular one [76,77]. The application of V-EMF therapy alone, even in the vacuum regime, could not be sufficient to regenerate the structures and restore functionality. For this reason, a marked weight loss was necessary to favor motor reactivation [78,79].

On the other hand, severe burns cause a pronounced metabolic response that leads to a hypermetabolic state, accompanied by severe catabolism and loss of lean body mass [52]. The metabolic stress response may last for years [80], causing a worsening of the body’s functional structure. Therefore, weight loss had to be preserved as much as possible, especially regarding the already weakened structure. An adequate nutritional choice could also ameliorate the regenerative effects, as already proven for peripheral nerve injury [81]. A very low-calorie diet (VLCD) and a very low-calorie ketogenic diet (VLCDKD) with amino acid supplements are nutritional programs that have both been shown to promote weight loss without inducing lean body mass loss and prevent the risk of sarcopenia [82,83,84].

In this case, the use of these types of nutritional programs has proven to be an effective solution to promote weight loss without impoverishing the structure and favoring the effects of the regenerative V-EMF therapy. Applying combined treatments in situations such as that described in this study seems to be the more successful choice for promoting and maintaining tissue regeneration. Among these treatments, nutritional strategies might play a fundamental role.

The initial situation of the woman described in this study perfectly illustrates the condition in which a person with major burns may find himself/herself after hospitalization. In the literature, the terms aftercare conditions and aftercare management are present [85]. Patients with severe burns often have residual deficits (functional and motor) [85,86,87] and must continue to treat their scars, especially with moisturizers, to avoid the shrinkage of the scar tissue or its degeneration into keloids [88]. Regarding motor deficits, rehabilitation is widely used but does not always allow complete recovery [85]. Regarding residual scars, if they are small, many options are available to reduce their size and thickness: laser and radiofrequency heating, microneedling, electroporation, and drugs [89]. However, high use of hydrating creams and sunscreens remains necessary for patients throughout their lives, and most of them also continue to wear pressure garments to reduce tactile fastidiousness [88]. In the case of extensive scars, these solutions are impracticable and total body therapeutic approaches, such as the one used in this study, are not described in the literature.

## 5. Conclusions

The results reported in this study, due to the combined application of electromagnetic and electrical stimulation in a vacuum regime and of a particular personalized nutritional program, are startling. A woman, covered by scars, with motor deficits that prevent her from living a normal life, as she was not able to walk without a walker, in less than one year, threw away the walker and ran.

This study reports only one case and documents in detail all the progress related to the application of the V-EMF therapy accompanied by a particular diet in this single case. Further studies on a larger number of patients are needed to generalize the results presented. However, if the reported results are consolidated with other analogous results, V-EMF therapy could become the elective therapy for severely burned patients.

## Figures and Tables

**Table 1:** Body composition parameters at different times.

Parameter	Normal Range	T0	T1	T2
Weight	53.5–72 kg	92 kg	83 kg	80 kg
BMI	18.5–24.9 kg/m^3^	31.8 kg/m^3^	28.7 kg/m^3^	27.7 kg/m^3^
Total Body Water (TBW)	46–57%	42.3%	48.4%	47.9%
Extra-Cellular Water (ECW)	40–48%	47.8%	-	43.6%
Intra-Cellular Water (ICW)	52–60%	52.2%	-	56.4%
Fat-Free Mass (FFM)	68–76.7%	57.5%	65.4%	65.4%
Fatty Mass (FM)	23.3–32%	42.5%	34.6%	34.6%
Body Cellular Mass (BCM)	40–70%	51.6%	-	56.2%
Mineral Mass (MM)	-	36.7%	-	44.9%
Skeletal Muscle Mass (SMM) Janssen	24.2–34.2%	25.2%	30.7%	29.6%
Appendicular Skeletal Muscle Mass (ASMM)	-	21.3 kg	21.3 kg	20.9 kg
Basic Metabolic Rate (BMR)	-	1541.7 kcal	1509.8 kcal	1602.6 kcal
Total Daily Energy Expenditure (TDEE)	-	1695.9 kcal	1660.8 kcal	1762.9 kcal

**Table 2:** Body circumferences before the treatment and after 6 months.

Site	T0	T2
Neck	39.5 cm	36.9 cm
Waist	121.4 cm	119.9 cm
Hips	125.6 cm	122.5 cm
Left Bicep	40.3 cm	40.5 cm
Right Bicep	43.2 cm	43.9 cm
Left Forearm	30.1 cm	28.7 cm
Right Forearm	32.5 cm	31.0 cm
Left Thigh	60.2 cm	58.6 cm
Right Thigh	61.2 cm	60.4 cm
Left Calf	41.5 cm	41.2 cm
Right Calf	42.5 cm	42.0 cm

**Table 3:** Lateral shift evaluated from the frontal view.

Site	Shift
Head	0.1 cm on the left
Shoulder	1.9 cm on the right
Underbust	1 cm on the right
Hip	0.7 cm on the right
Knee	0.8 cm on the right

**Table 4:** Body projection evaluated from the lateral view.

Site	Shift
Head	8.4 cm of forward projection
Shoulder	6.4 cm of forward projection
Hip	4.3 cm of forward projection
Knee	3.1 cm of forward projection

## Data Availability

All the data used for this study are present in the text.
